# Inflammation and immune cell abnormalities in intracranial aneurysm subarachnoid hemorrhage (SAH): Relevant signaling pathways and therapeutic strategies

**DOI:** 10.3389/fimmu.2022.1027756

**Published:** 2022-11-23

**Authors:** Jing Jin, Jian Duan, Leiya Du, Wenli Xing, Xingchen Peng, Qijie Zhao

**Affiliations:** ^1^ Department of Pharmacy, West China Hospital, Sichuan University, Chengdu, Sichuan, China; ^2^ Department of Biotherapy, Cancer Center, West China Hospital, Sichuan University, Chengdu, Sichuan, China; ^3^ Department of Cerebrovascular Disease, Suining Central Hospital, Suining, Sichuan, China; ^4^ 4Department of Oncology, The Second People Hospital of Yibin, Yibin, Sichuan, China

**Keywords:** subarachnoid hemorrhage (SAH), inflammation, immune cells, signaling pathways, therapeutic strategies

## Abstract

Intracranial aneurysm subarachnoid hemorrhage (SAH) is a cerebrovascular disorder associated with high overall mortality. Currently, the underlying mechanisms of pathological reaction after aneurysm rupture are still unclear, especially in the immune microenvironment, inflammation, and relevant signaling pathways. SAH-induced immune cell population alteration, immune inflammatory signaling pathway activation, and active substance generation are associated with pro-inflammatory cytokines, immunosuppression, and brain injury. Crosstalk between immune disorders and hyperactivation of inflammatory signals aggravated the devastating consequences of brain injury and cerebral vasospasm and increased the risk of infection. In this review, we discussed the role of inflammation and immune cell responses in the occurrence and development of aneurysm SAH, as well as the most relevant immune inflammatory signaling pathways [PI3K/Akt, extracellular signal-regulated kinase (ERK), hypoxia-inducible factor-1α (HIF-1α), STAT, SIRT, mammalian target of rapamycin (mTOR), NLRP3, TLR4/nuclear factor-κB (NF-κB), and Keap1/nuclear factor (erythroid-derived 2)-like 2 (Nrf2)/ARE cascades] and biomarkers in aneurysm SAH. In addition, we also summarized potential therapeutic drugs targeting the aneurysm SAH immune inflammatory responses, such as nimodipine, dexmedetomidine (DEX), fingolimod, and genomic variation-related aneurysm prophylactic agent sunitinib. The intervention of immune inflammatory responses and immune microenvironment significantly reduces the secondary brain injury, thereby improving the prognosis of patients admitted to SAH. Future studies should focus on exploring potential immune inflammatory mechanisms and developing additional therapeutic strategies for precise aneurysm SAH immune inflammatory regulation and genomic variants associated with aneurysm formation.

## Introduction

Intracranial aneurysms are common and have a high incidence of occurring in 1% to 2% of the population, wherein the incidence of rupture is nearly 16.4 in 100,000 persons per year (1, 2). Subarachnoid hemorrhage (SAH) is a serious clinical condition that is usually caused by a ruptured intracranial aneurysm, which caused nearly 85% of SAH (3, 4). SAH was reported to occur at a fairly young age, and the mortality rate of aneurysmal hemorrhage is nearly 50% (5). Numerous clinical trials have been conducted to improve outcomes for patients with SAH, whereas there are still challenges in the aneurysm SAH prevention and lower-risk treatment development (4).

SAH can be separated into traumatic and spontaneous, where the spontaneous SAH is known to have the highest incidence and is most often attributed to a ruptured intracranial aneurysm (6). Cerebral aneurysm-acquired lesions that develop at the major arterial branch point of the Willis circle result in hemodynamic stress-induced retrogradation of the internal elastic lamina with loss of the tunica media (4). Intracranial aneurysm SAH is a critical cerebrovascular accident with high mortality and high disability among survivors (7). Some pathophysiological factors are independent of angiographic vasospasm and are related to poor clinical prognosis, such as blood–brain barrier (BBB) disruption, inflammation, immune cell activation, and oxidative cascades, ultimately contributing to cell death (8, 9). Among which milieu, microglial-induced immune responses like macrophage were positively associated with neuroinflammation development and neuronal necrosis after SAH (10, 11). Injured neurons and dying cells will release inflammatory molecules to the extracellular milieu, which was associated with poor clinical outcomes in patients with aneurysm SAH (12, 13). These dangerous molecules may further drive the neuroinflammation and brain injury after SAH (14). Brain injury following intracranial aneurysm SAH is multimodal and serious, as early brain injury (EBI), but is also secondary to the development of immune-inflammation events (9, 10, 15, 16). Crosstalk between immune cell populations, active substances, and inflammation responses may aggravate the symptoms of SAH and contribute to poor prognosis (9, 17, 18). Treatment of immune-inflammation disorders has great potential to attenuate EBI and devastating secondary damage and ameliorate outcomes in patients with SAH. Therefore, the identification of immune-inflammation mechanisms of intracranial aneurysm SAH and its associated sequelae could be beneficial for these patients (19).

In the past decade, several treatable risk factors (cigarettes, alcohol, hypertension) and untreatable risk factors (age, sex, genetics) have been reported to increase the incidence of aneurysms (2, 20). The mechanisms of intracranial aneurysm occurrence and rupture are complex, especially immune microenvironmental and genetic factors (21). Abundant evidence supported that the etiology of intracranial aneurysms is related to genetic factors (22, 23). Genetic syndromes associated with intracranial aneurysms have been identified as an increased risk compared with the general population (2, 21). Meanwhile, individual genetic variations proposed higher aneurysm SAH and worse neuronal injury, such as THSD1 and EDN1 gene variants which were highly enriched in aneurysm SAH patients (24, 25). Specific biomarkers for intracranial aneurysm provide a potential therapeutic avenue for intracranial aneurysms, such as platelet-derived growth factor receptor β gene (PDGFRB) (26). Despite the knowledge of genetic and inflammatory mechanisms of brain injury caused by intracranial aneurysm SAH which is currently understood, the complexity of the immune cell responses and the crosstalk of the above factors in this process have not been described in detail.

In this review, we summarized the immune cells and inflammation-related mechanisms during the occurrence and development of aneurysm SAH, as well as several pivotal signaling pathways related to immune inflammatory, vasospasm, EBI, and therapeutic potential after aneurysm SAH. In addition, we discussed potential therapeutic drugs targeting the immune and inflammatory response, as well as prophylactic agents for aneurysm SAH. Understanding the specific pathological mechanisms of aneurysm SAH is important for developing strategies to prevent disease development and brain injury.

## The intracranial and subarachnoid hemorrhages

Intracranial hemorrhage (ICH) refers to any bleeding within the intracranial vault, such as brain parenchyma and meningeal spaces (27). SAH is regarded as bleeding into the space between the pia and the arachnoid membranes (28). Non-traumatic causes of hemorrhages include ruptured aneurysms, arteriovenous malformations, tumors, and vasculopathies (29–31). Previously, brain tumors like glioma/glioblastoma have a direct compressive or invasive function on the cerebral vessel, and it is observed to have a high incidence of ICH and fatal outcomes (32, 33). In both primary or metastatic brain tumors with ICH, angiogenesis mediators vascular endothelial growth factor (VEGF) and matrix metalloproteinases (MMPs) were associated with vascular rupture hemorrhage (31). More recently, in acute leukemia-related ICH, early ICH is characterized by leukostasis associated with abnormal hemostasis, whereas late ICH has systemic inflammation (34). On the other hand, aneurysmal SAH is due to rupture of an aneurysm in the subarachnoid space, which is commonly seen at the bifurcation of the basal cerebral artery, especially near the circle of Willis (35, 36). To date, the risk factors of aneurysmal growth and rupture remain complex; for example, size (>7 mm), inflammation, genetic syndromes, and hypertension can contribute to the aneurysmal rupture and SAH (37, 38).

For ICH, secondary brain injury following ICH is closely associated with hematoma toxicity, oxidative stress, and inflammation, among which hematoma toxicity and oxidative stress are mediators of cell death (39–41). Aronowski et al. indicated that hematoma will contribute to direct mechanical injury to the brain parenchyma, as well as perihematomal edema (42). The porphyrin derivatives were observed to inhibit heme oxygenase 1 (HO-1) and reduce the ICH damage (43). HO-1, an enzyme involved in biliverdin, carbon monoxide, and iron conversion (44), was observed with an increase in endothelial cells and microglial/macrophages after ICH (45). Of note, HO-1 deficiency mice showed ameliorated ICH-mediated brain damage, which was different from the ability to aggravate injury in many other brain injury models (44). Recently, in this aspect, low HO-1 expression in early SAH patients has been associated with vasospasm, whereas delayed cerebral ischemia (DCI) showed higher HO-1 levels (46, 47). In addition, under ICH pathological status, an overproduction of reactive oxygen species (ROS) was observed, where bivalent iron (Fe^2+^) promotes hydrogen peroxide (H_2_O_2_) disintegration (39, 48) and oxidase enzyme participates in the ROS biological generation process (41, 49). Meanwhile, mice with a generically deleted NADPH enzyme showed reduced damage after ICH (41). Recently, ROS accumulation after SAH has been considered to be a by-product of oxidative phosphorylation in the mitochondria, which is a major target of ROS-induced damage in SAH patients (50). Similarly, both early stages of SAH and ICH were accompanied by ROS generation, which impaired antioxidant defense systems and signal cascade responses (49, 51, 52).

Hematoma formation after ICH usually stimulates inflammatory reaction through microglial/macrophages and/or inflammatory signaling pathways, thereby contributing to immune cascade activation and pro-inflammatory cytokine secretion (53–55). Activated microglia were previously reported to recruit hematogenous inflammatory cells to the ICH injury areas by cytokines and chemotactic factors (56). Meanwhile, microglial/macrophage-mediated phagocytosis facilitates brain cleanup after the early inflammatory responses after ICH, where multicellular surface receptors (CD36, CD91, and SLC) assist in reducing cellular debris following ICH (57, 58). With inflammatory signaling coordination, oxidative stress can enhance the inflammation response after ICH, such as nuclear factor-κB (NF-κB), TNFα, and matrix metalloproteinase-9 (MMP-9) (42). In the chronic phase of ICH, inflammatory stress will impair the white matter tracts and contribute to severe neurological dysfunctions, especially motor and memory functions (59). Evidence indicated that NF-κB is activated in ICH-related brain injury as early as 15 min after the hemorrhage, which will induce nitric oxide synthase (iNOS), TNFα, interleukin, and cyclooxygenase-2 inflammatory cytokines (60, 61). Expression of these genes will lead to neuroinflammation and BBB hyperpermeability (62).

## SAH-related inflammation responses

**Figure 1:**
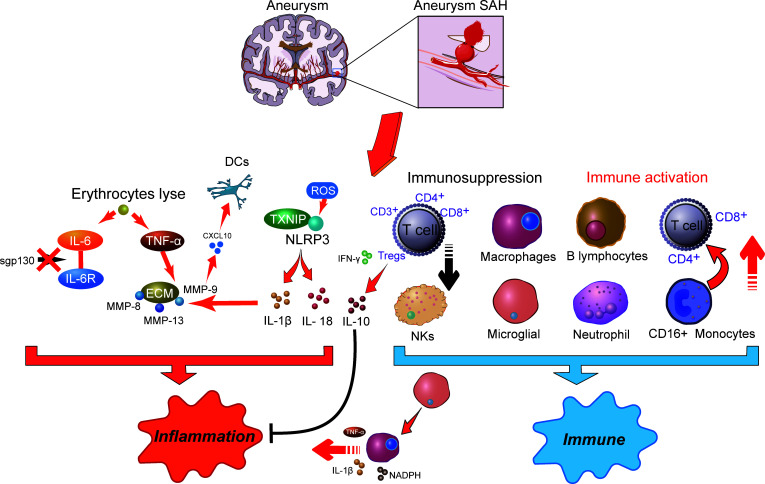
The potential molecular mechanisms of inflammatory effects (right) and immune responses (left) in aneurysm-related subarachnoid hemorrhage. SAH, subarachnoid hemorrhage; DCs, dendritic cells; IL-6, interleukin 6; ROS, reactive oxygen species; MMP, matrix metalloproteinases; NLRP3, NOD-like receptor family pyrin domain-containing 3; TXNIP, thioredoxin-interacting protein; TNF-α, tumor necrosis factor-α; INF-γ, interferon-γ; NKs, natural killer cells; NADPH, nicotinamide adenine dinucleotide phosphate oxidase; Tregs, regulatory T cells.

Inflammation is correlated with various neurodegenerative diseases, including SAH, Alzheimer’s disease, and Parkinson’s disease (63). Inflammation is an important mechanism that has been implicated in the pathogenesis of SAH, where cellular inflammation- and molecular inflammation-elicited neuronal injuries have been detected in the subarachnoid space (15). Innate cell immunity obviously generates inflammation responses in the subarachnoid space in an inside-out form. Molecular agents of inflammation were proposed to be increased within posthemorrhagic aneurysms (**Figure 1**), where factors such as IL-6 and TNF-α are correlated with poor clinical prognosis (15, 19). In addition, within brain injury and the related inflammatory responses, lysis of erythrocytes after SAH showed a positive correlation with increased levels of IL-6 and TNF-α in the brain cortex (64). Following aneurysmal SAH, the increase in pro-inflammatory cytokine IL-6 has been recently presented with neutrophil accumulation in the brain and local and peripheral inflammation responses (65, 66). Immune cell infiltration showed a target therapeutic potential in patients with aneurysmal SAH (65). Moreover, IL-6 has been further defined as a contributing factor to brain injury and is related to poor clinical prognosis (67), wherein IL-6 involved in neuroinflammation response is closely associated with EBI after aneurysmal SAH (68–70). Of note, soluble gp130 (sgp130) and IL-6 receptor (IL-6R) represented the IL-6-transducing antagonist and agonist receptors, respectively (71). With the development of SAH, the level of the IL-6 antagonist gp130 is increased to antagonize the elevated levels of IL-6, which decreases within a few days, presumably resulting in cerebral vasospasm and neuroinflammatory injury (71). Recent studies showed that full-length gp130 is the most potent inhibitor of IL-6 trans-signaling (72). Most recently, a study has shown that through IL-6 signaling, tissue-specific sgp130 can trigger the upregulation of innate immune system components (73), where sgp130-related immune cell and chemokine recruitment might protect against neuroinflammation (74, 75). The molecular weight of the main sgp130 isoforms ranges from 50 to 110 kDa, which has a high affinity (1 mM) for the IL-6/IL-6R complex to neutralize its pro-inflammatory functions (76).

Although numerous histochemical alterations occur during the development of an aneurysm up to the point of rupture, the release of blood into the subarachnoid space afterward contributes to more serious histological and inflammatory changes (77). Another important inflammation activation-related element, thioredoxin-interacting protein (TXNIP) that interacts with the NOD-like receptor family pyrin domain-containing 3 (NLRP3) inflammasome to induce interleukin IL-1β secretion, was previously demonstrated to connect with tumorigenesis and insulin resistance (78). Meanwhile, inflammasomes are part of the innate immune system. The NLRP3 inflammasome is a multiprotein complex that orchestrates innate immune responses, whereas unregulated NLRP3 inflammasome activation in pathology responses can lead to unintended immune and inflammatory pathological conditions, such as mitochondrial metabolism and ROS accumulation (79). Recently, the NLRP3 inflammasome has been extensively studied and observed to be associated with the release of IL-1β and IL-18, which exacerbated the inflammation response after SAH and promoted the occurrence of EBI (80, 81). The intervention of the NLRP3 signaling cascade can alleviate neuroinflammatory responses and restore neurobehavioral function (82). NLRP3 activators can produce ROS, which subsequently activate the inflammasome (83). A strong antioxidant melatonin has been shown to protect against EBI and inflammatory response after SAH (84) and to improve aneurysm SAH clinical outcomes (85). Among which, melatonin suppressed pro-inflammatory cytokine levels in the cortical levels, such as IL-1β, IL-6, and TNF-α (84). Upregulation of these cytokines has been demonstrated to exacerbate brain disorders after SAH (86).

In addition, during aneurysm rupture and consequent SAH, extracellular matrix (ECM) remodeling plays an important role in inflammation. TNF-α has been demonstrated as an upstream regulator for MMP-9 (87). MMP gene expression is upregulated after SAH, where MMP-8, MMP-9, and MMP-13 were observed to accumulate in the vascular wall *via* the p38 kinase signaling pathways (88). Among them, MMP-9 has been recently reported to have a consistently higher level in aneurysm SAH patients, which may cause cerebral vasospasm, DCI, and neuronal death by promoting neuroinflammation (89). MMPs also participate in the inflammation regulation of pro-inflammatory cytokines and chemokines, especially the function of MMP-9 on CXCL10 and CCL2 (90, 91). Of note, CXCL10 is associated with intrathecal immune activation and dendritic cell (DC) accumulation after aneurysm SAH (92). On the other hand, MMPs are a family of zinc endopeptidases that can open the BBB by degrading tight-junction proteins (93). Melatonin treatment reduces VEGF expression to prevent BBB disruption following SAH (84). Furthermore, VEGF is modulated by several extracellular stimuli, including pro-inflammatory cytokines like IL-6 and TNF-α (94). IL-1β is also an important regulator of MMP-9 and causes BBB disruption after SAH (95). Thus, a variety of immune inflammation processes occur in different compartments following aneurysm SAH and are possibly associated with inflammatory cytokines and immunomodulatory molecule generation.

## Immune cell abnormality in SAH

Although accumulating evidence supports the function of inflammation in aneurysmal SAH, the exact immune mechanisms remain to be elucidated. It has been postulated that SAH following aneurysm rupture induces immune responses including secretion of active substances with vasoactive and pro-inflammatory functions, ultimately contributing to EBI (18, 96–98). Immunosuppression following nervous system injury is a critical issue clinically (**Figure 1**), because more than 50% of brain-injury patients develop infection (99). In symptomatic aneurysmal SAH patients, poor outcome is associated with symptoms of impaired local immune competence (100). Substantial evidence suggested that temporary impairment of the immune system is an important risk factor in the emergence of infection after aneurysmal SAH (9). Furthermore, pronounced SAH-induced immunosuppression is detected in the early stages of injury after aneurysmal SAH, where a reversed correlation between IL-6 level and CD3^+^ T cells was observed (101). Among which, the high incidence of bacterial pneumonia in symptomatic aneurysmal SAH patients may be attributed to impaired immune responses and reduced T-cell count. In a previous study, clinical investigations suggested that the risk of subsequent SAH was associated with immune-mediated diseases, such as autoimmune hemolytic anemia, Crohn’s disease, and hyperthyroid conditions (102). In addition, patients with aneurysmal SAH undergoing surgical treatment experienced a transient deterioration in immune functions, especially immunosuppression (9). Decreased immune cell subgroups were significantly associated with aneurysmal SAH, such as the downregulation of CD3^+^, CD4^+^, CD8^+^ T cells, natural killer cells (NKs), and regulatory T cells (Tregs), leading to an unfavorable postoperative prognosis. Nevertheless, following stroke, activated T cells infiltrated the brain, consequently releasing cytokines and ROS, which may result in brain injury, where ROS likely contributed to neuronal inflammation, neuronal cell death, and poor outcomes (103). After that, increased neuroantigens could further induce adaptive immune response and cause additional T-cell activation and brain injury. However, in the middle/late stages in DCI patients, aneurysm SAH-induced immunosuppression was observed to decrease the T-cell population, resulting in an increased risk of infectious complications (103, 104). In a more recent study, following aneurysm SAH, immunosuppressive Tregs were significantly increased and presented a different activation status in the EBI and DCI phases (105). In patients with DCI, CD3^+^ Tregs showed a higher population compared with EBI and were closely associated with infections. Meanwhile, CD3^-^ Tregs were significantly reduced in patients with EBI. In the EBI phase, low-dose IL-2 treatment significantly prevented the Treg population and suppressed neuroinflammation following SAH, wherein the decreased proinflammatory factors and peripheral neutrophils improved neuronal injury and neurological functions (106). Plausibly, activated Tregs have the effective ability to inhibit the conventional T-cell proliferation and readily produce cytokines (107). Herein, under these circumstances, immunosuppressive Tregs act as modulators of the immune system, resulting in suppression of inflammation by affecting the pro-inflammatory (TNF-α and IFN-γ) and anti-inflammatory (IL-10) factor generation (108–110). Moreover, Tregs also suppress the peripheral MMP-9 production, thereby preventing BBB damage and neuroinflammation (111), which showed the neuroprotective effect and therapeutic potential for aneurysm SAH.

On the other hand, immune activation after aneurysmal SAH has been shown to play a pivotal role in host defense against infection (9). Shortly after the aneurysmal rupture, damage to the brain tissue and blood components led to the exposure of antigens that stimulated innate immune function, which might contribute to its activation and induction of acute immune-inflammatory responses (112). Subsequently, innate immune responses generate molecules that deliver signals, resulting in activation of T cells, effector cells, and B lymphocytes to attach in proinflammatory vessels with release of various adhesion factors and cytokines (112–114). The release of pro-inflammatory cytokines directly eliminates damaged cells, induces and regulates inflammation, and destroys microbes (104). Moreover, the increase in the M1/M2 macrophage ratio plays an important role in both intracranial aneurysm and SAH (115, 116). CXCL1 antibody intervention may give potential to increase the macrophage proportion and anti-inflammatory function. Macrophages can eliminate dead cells and debris and provide defense against infection (117, 118), which will decrease the EBI and complications in aneurysm SAH (119). However, M1 polarization of macrophages is a proinflammatory phenotype associated with reduced debris removal capability and enhanced production of proinflammatory cytokines like TNFα, IL-1β, and NADPH, ultimately contributing to nervous system inflammation (120). It is becoming increasingly clear that the dual role of immune cells needs to be further explored.

Previously, Balboa et al. reported that high levels of CD16^+^ monocytes stimulated T-cell proliferation and predicted higher antigen-presenting cell (APC) activity in peripheral blood (PB) (121). Indeed, a threefold increase in the potency of APCs was observed in CD16^+^ monocytes compared with CD16^−^ monocytes (122). Recently, PB analysis in aneurysm SAH patients observed the activation of some immune cell subpopulations such as CD4^+^/CD8^+^ T cells, CD16^+^ monocytes, and neutrophils (123). These results highlight the participation of innate immunity in aneurysmal SAH. The increased proportion of CD16^+^ monocytes potentially indicated that stimulation of the innate immune system was resided in aneurysmal SAH patients. In addition, the expression of cell-based CD28 in the adaptive immune system induced greater activation of aneurysmal SAH CD4^+^ and CD8^+^ T cells in PB than in the cerebrospinal fluid (CSF) (123). CD28 is also the B7 receptor expressed on naïve T cells and provides costimulatory signals that are required for T-cell activation (124). Moreover, CD28 stimulation induces T-cell activation of potential co-stimulatory signals, consequently leading to the generation of various interleukins (125). In terms of this, increased IL-2 receptor and CD8 levels in SAH patients have shown the vital function of immune response in SAH pathogenesis (126). Thus, these findings indicated the participation of innate and adaptive immune responses in the immunopathogenesis of aneurysmal SAH.

The results of pilot studies may be various, but distinguishing the mechanisms of immune suppression and hyperactivation will facilitate the provision of personalized patient treatment to regulate the immune function and protect against aneurysmal SAH. Dysregulation of the immune cell subgroup is closely associated with the clinical prognosis of aneurysmal SAH patients (101, 123), which might be a candidate biomarker to predict patient diagnosis as well as the development of effective therapeutic strategies to eliminate the complications in aneurysmal SAH. Further research on the immunosuppression induced by aneurysm SAH (especially Tregs) and its relationship with inflammatory factors can provide new ideas for the treatment of aneurysm SAH.

## Immune inflammation relevant signaling pathways in SAH

Signaling pathway dysfunction can lead to poor outcomes after aneurysmal SAH, which is closely related to primary and secondary injuries in disease development. A precise signaling pathway regulation that triggers both immune modulation and inflammatory responses hold great promise in elucidating pathological mechanisms after SAH. A more comprehensive understanding of SAH-related immune inflammation underlying mechanisms will boost our ability to develop novel therapeutic options. Herein, based on current knowledge, we discussed immune cell and inflammatory function relevant signaling pathway modulation in the context of aneurysmal SAH (**Table 1**).

**Table 1:** Different pathways relevant modulators and effects in aneurysm SAH.

Pathway	Modulator	Relationship	Immune cells and/or cytokines	Relevant effects	References
PI3K/Akt	Aggf1	Positive	Microglia and neutrophil	Improve the inflammatory response; alleviate secondary brain injury.	(127, 128)
	MFG-E8, CXCL12	Positive	//	Promote vascular endothelial repair.	(129)
	LRP1, SHC1, pSer473	Positive	M2 microglial	Improve white matter injury and inflammation.	(130–134)
	RARα, IKKα/β	Positive	M2 microglial	Improve inflammatory response and reduce neuronal apoptosis after SAH.	(135–137)
	EAAT2	Positive	Astrocytes	Improve EBI and immune responses after SAH.	(138–140)
	TNC	Negative	//	Contribute to neuroinflammation.	(141)
	5-lipoxygenase	Negative	LTB4, TNF-α, IL-1β, and IL-6	Contribute to EBI after SAH.	(142)
ERK	Raf proteins	Positive	IL-6, IL-1β, and MMP-9	Promote inflammatory response.	(143)
	Compound C	Positive	Microglial	Promote neuroprotection.	(144)
	Peli1	Positive	M1 microglia	Contribute to neuroinflammation in EBI following SAH.	(145)
	LXA4	Positive	TNF-α, IL-1β, and IL-6	Improve inflammatory response after SAH.	(146)
HIF-1α	TLR4	Positive	TNF-α and interleukin	Promote inflammatory response after SAH.	(147)
	2-ME	negative	Microglia, IL-1β, IL-6, and TNF-α	Improve inflammatory response and EBI.	(148)
STAT	HMGB1	Positive	IL-1 and MMP-9	Promote inflammation EBI after SAH.	(149–151)
	NOX2	Positive	M1 microglia	Promote the oxidative stress and inflammation.	(152)
	PK2	Positive	A2 astrocytic	Improve immune and inflammation environment to alleviate EBI after SAH.	(153, 154)
	EPO receptor	Positive	M2 microglial	Alleviate inflammation.	(155)
	TSG-6	Negative	Microglia/macrophages	Protect immune cell and alleviate inflammation.	(156)
SIRT1	RSV	Positive	IL-1β, IL-6, and TNF-α	Improve inflammation.	(157)
	MR	Positive	NF-κB	Improve EBI after SAH.	(158)
	PDE-4	Negative	Microglia, IL-10, TNF-α, IL-1β, and IL-6	Promote SAH-induced EBI.	(159, 160)
	HMGB1	Negative		Promote inflammatory response.	(161)
	OA	Positive	TLR4, TNF-α, IL-1β, and NF-κB	Improve inflammation.	(162)
	NLRP3	Negative	IL-1β	Promote inflammatory response and aneurysm rupture.	(163)
TGF-β	CB2R	Positive	TGF-β1 and E-selectin	Prevent leukocyte infiltration and BBB after SAH.	(164)
	GPR120	positive	TAK1	Improve inflammation.	(165, 166)
mTOR	P70S6K1, 4E-BP1	Positive	//	Promote cerebral vasospasm after SAH.	(167, 168)
	beclin-1	Negative	//	Promote neuroprotective effects.	(169)
TLR4/NF-κB	MCP-1	Positive	Macrophages	Promote inflammation.	(170)
	NFKBIA	Negative	//	Improve inflammation and apoptosis.	(171)
	Prx2	Positive	Microglia	Promote inflammation.	(172)
Keap1/Nrf2/ARE	NF-κB p65	Negative	Astrocytes	Promote inflammation.	(173–175)
	PHB2	Positive	//	Improve EBI after SAH.	(7, 176)

### The PI3K/Akt signaling pathway

**Figure 2:**
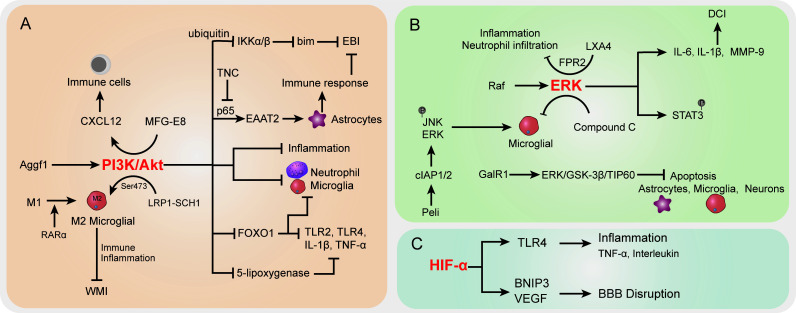
The specific role played by PI3K/Akt **(A)**, ERK **(B)**, and HIF-α **(C)** pathways in disease development following SAH. CI, delayed cerebral ischemia; EBI, early brain injury; BBB, blood–brain barrier; WMI, white matter injury.

PI3K/Akt signaling pathway dysregulation was previously demonstrated to be associated with various SAH sequela, such as EBI, vasospasm, and neurological injury. The onset of the Akt cascade is activated by tyrosine kinases, immune cell receptors, cytokine receptors, G-protein-coupled receptors, and stimulation of PIP3 generation by PI3K that potentially further influences the immune inflammatory response (177, 178). In SAH, the upregulated Aggf1 expression will provoke the PI3K/Akt signaling and decrease upstream NF-κB activation to improve the inflammation response (127) (**Figure 2A**). Of note, the above interactions were presented with decreased neutrophil infiltration and microglial activation. The suppression of neutrophils showed potential to improve the immunosuppression response by decreased immune cell monocyte recruitment, thus alleviating secondary brain injury (128). On the other hand, proper immune boosting may also be beneficial in protecting nerves in the brain. The CXCL12 chemotaxis for T cells, lymphocytes, and macrophages had previously been considered to maintain the immune environment in injured blood vessels (179) and played a pivotal role in neuroprotection and against neuroinflammation in recent studies (180, 181). Moreover, Wang et al. indicated that milk fat globule–epidermal growth factor 8 (MFG-E8) exhibited vascular endothelium protection effects through promoting the PI3K/Akt/CXCL12 cascade (129). In the brain of SAH, MFG-E8 directly enhanced PI3K expression and CXCL12 to promote vascular endothelial repair, wherein PI3K activation is causative for increased CXCL12 expression (129). However, the underlying mechanisms of immune cell regulation remain obscure. Furthermore, low-density lipoprotein receptor-related protein-1 (LRP1) activation was reported to attenuate white matter injury (WMI) in SAH patients *via* the PI3K/Akt pathway, wherein the intracellular adaptor protein SHC1 was required for LRP1 transduction (130). M2 microglial polarization was found to be associated with inflammation-induced functions, and the LPR1 ligand mediated anti-inflammatory M2 microglial phenotypes after SAH (130, 131). The morphological changes of microglia, as the immune cells of the brain, are closely related to their functions (132). Importantly, Akt was appeared to play a crucial role in M1 to M2 polarization *via* regulation of Ser473 phosphorylation after WMI (133). The activated microglia do not merely modulate the endogenous immune response of brain injury but also alleviate inflammation (134). Similarly, retinoic acid receptor α (RARα) was demonstrated to promote M1 to M2 microglial phenotypic polarization and has anti-inflammatory effects after SAH, relying mainly on regulating the PI3K/Akt pathway (135). The activation of Akt was involved in the phosphorylation of the inflammation-related proteins IKKα/β, through activating the ubiquitin/protease system to promote IKK degradation (136). As a result, this cascade is shown to reduce neuronal apoptosis after SAH (136). On the other hand, the inhibition of PI3K was accompanied by an elevated bim protein level, which is important for cell apoptosis (137). In the mouse model, the bim gene was found to be regulated by IKK and was positively associated with SAH-induced EBI (182, 183).

In more downstream candidates of the PI3K/Akt cascade, the transcription factor forkhead box protein o1 (FOXO1) was negatively associated with PI3K/Akt signaling activation; it showed the ability to regulate downstream pro-inflammatory molecules (TLR2, TLR4, IL-1β, and TNF-α) and different types of immune cells (neutrophils, macrophages, DCs, and Tregs) (184). Whether the decrease in FOXO1 protein here affects immune response in SAH needs further analysis. Intriguingly, the EBI after SAH was characterized by the reduction of EAAT2 in astrocytes, which was directly regulated by Akt signaling (138). Data indicated that excitatory amino acid transporter 2 (EAAT2) deficiency in astrocytes was closely associated with innate and adaptive immune pathway disorder. In a mouse model of SAH, Akt activity was observed to be decreased in the brain, thereby leading to a lower EAAT2 level (138), whereas the reactivation of Akt signaling will promote the p65 phosphorylation and significantly improve EAAT2 expression in astrocytes (139, 140), ultimately ameliorating EBI after SAH. The activation of the PI3K/Akt signaling pathway represents a promising positive effect on EBI after SAH (185), as well as a neuroprotective effect (186, 187). In an oxygen hemoglobin-induced SAH mouse model, the upregulated tenascin-C (TNC) after SAH impaired the PI3K/Akt/p65 cascade, thereby leading to neuroinflammation (141). Moreover, the PI3K/Akt cascade was reported to participate in the alleviation of inflammation by inhibiting inflammatory mediators in stroke and promoting tight-junction proteins to protect BBB integrity (188, 189). In terms of neuroinflammation and BBB disruption caused by SAH, PI3K/Akt cascade activation attenuated the above symptoms (127). Recently, Liu et al. reported that increased 5-lipoxygenase in cytoplasm of cortical neurons along with expression of upregulated inflammatory factors LTB4, TNF-α, IL-1β, and IL-6 contributed to EBI after SAH (142). In this process, activation of PI3K/Akt signaling significantly suppressed the 5-lipoxygenase-induced SAH pathologic manifestation (142). Taken together, the activation of the PI3K/Akt signaling pathway potentially improves the immune inflammatory response, eventually resulting in protection against the damage following SAH.

### ERK signaling pathway

The Raf-mitogen-activated protein kinase kinase (MEK)1/2-extracellular signal-regulated kinase (ERK) 1/2 pathway is one of the components of six MAPK signal transduction pathways that are widely involved in cell regulation (190) (**Figure 2B**). SAH increased P38 MAPK phosphorylation and attenuated the phosphorylation of ERK (191). Phosphorylation proteomic analysis suggested that the STAT3 pathway was activated upon SAH induction, most likely downstream of ERK1/2, because STAT3 phosphorylation was suppressed by MEK1/2 inhibition (192). Transcriptional overexpression of inflammatory molecules (cytokines and metalloproteinases) in cerebral arteries is caused by SAH-induced activation of the MEK/ERK pathway (143, 193). According to a previous study, cytokine (IL-6 and IL-1β) and MMP-9 upregulation can be prevented by specific blockade of the MEK/ERK pathway *via* inhibiting upstream Raf proteins after SAH (143), indicating that the MEK/ERK pathway plays a crucial role in DCI following SAH and the cerebrovascular inflammatory response. Synchronously, another research by Maddahi et al. indicated that inhibition of the MEK1/2 pathway only within the time window of 6–24 h after SAH can change cerebrovascular inflammatory response and neurological prognosis later following SAH (194). The underlying mechanism is that IL-1β, IL-6, MMP-9, and pERK1/2 protein expression levels in cerebral artery walls increased with time and increased at the early stage of 6 h after SAH and reached the peak at the late stage of 48–72 h. At the early time points (1 to 24 h) post-SAH, TNFα immunoreactivity in the brain tissue was remarkably enhanced, which is colocalized with glial fibrillary acidic protein (GFAP), a marker of astrocytes and glial cells in perivascular and brain tissues (194).

In addition, the effect of compound C (a classical inhibitor of MAPK) on microglial shape change was mediated by activated ERK1/2, PI3K/Akt signaling, or small Rho GTPase, which provided evidence for the neuroprotective role of compound C in SAH (144). As a clue, Peli is an adaptor protein that interacts with Pelle, which is a Drosophila homologue of the mammalian interleukin-1 receptor-associated kinase (195), whereas, as an E3 ubiquitin ligase, Peli was also upregulated in TLR4-dependent microglial activation post-SAH in a time-dependent manner and induced proinflammatory cytokine IL-6 in microglia (145, 196). Peli1 induced microglia-mediated neuroinflammation in EBI following SAH by enhancing the phosphorylation levels of ERK and JNK *via* cIAP1/2 activation. Meanwhile, Peli1 also encouraged M1 microglia to exhibit the polarization markers CD16/32 and iNOS after SAH, indicating that the inhibition of Peli1 might generate neuroprotective effects during EBI after SAH (145). Following SAH, the expression of lipoxin A4 (LXA4), an important endogenous lipid, is suppressed, whereas pro-inflammatory cytokine (TNF-α, IL-1β, IL-6) and factor (NF-κB, MMP9, ICAM-1, MPO) expressions were upregulated. Application of LXA4 in mice after SAH attenuates the above inflammatory response and neutrophil infiltration through the LXA4/FPR2/ERK1/2 signaling pathway (146). Moreover, EBI after SAH has been proved to be significantly pathologically influenced by neuronal apoptosis in pathological aspects (197). Activation of galanin receptor 1 (GalR1) has an anti-apoptotic effect in ischemic stroke. More recently, Shi et al. indicated that GalR1 is expressed in some astrocytes and microglia, but mainly in neurons, and activation of GalR1 is reported to recede neuronal apoptosis *via* the ERK/GSK-3β/TIP60 pathway after SAH (198). In summary, the ERK pathway plays an important role in inflammatory response after SAH, and early inhibition of ERK signaling after SAH may be effective in neuroprotection.

### The HIF-1α signaling pathway

In the context of ischemic stroke and cerebral hemorrhage, hypoxia-inducible factor-1 (HIF-1) is reported to have a dual function by stimulating both pro-survival and pro-death pathways in the central nervous system (CNS) (199, 200) (**Figure 2C**). HIF-1 protein expression was upregulated at 12 h and reached the peak at 24 h after SAH (201), and HIF-1 stimulation may be detrimental at an early stage after SAH, whereas activation of HIF-1 could be neuroprotective at a later stage post-SAH, suggesting that HIF-1 also performs pro-survival and pro-death roles following SAH (202). In a rat model of SAH utilizing endovascular perforation, HIF-1α can cause cell apoptosis, BBB disruption, and brain edema in EBI after SAH by upregulating the activation of BNIP3 and VEGF expression (203, 204). Simultaneously, as a target of miR-675, HIF-1α improved TLR4 expression *via* increasing the TLR4 promoter’s transcriptional activity, whereas TLR4 is essential for pro-inflammatory cytokine (TNF-α and interleukin) release following SAH. Nevertheless, these pro-inflammatory cytokines participate in cell apoptosis which play a crucial manifestation of post-SAH EBI (205). In addition, 2-methoxyestradiol (2-ME), a natural endogenous metabolite of 17-β estradiol, has antitumor, anti-angiogenic, and anti-inflammatory abilities (147, 206). Research data showed that 2-ME can reduce inflammatory factor (IL-1β, IL-6, and TNF-α) expression levels; downregulate brain water content, microglial activation, BBB permeability, and cell apoptosis; and enhance neurological dysfunction in rats. However, the mechanism of this protective effect is that 2-ME inhibits the expressions of HIF-1α, MMP-9, and VEGF, which is related to BBB disruption after SAH and inflammatory response to EBI (148). The HIF-1α signaling pathway as a regulatory target of inflammatory response after SAH needs to be further investigated.

### The STAT signaling pathway

**Figure 3:**
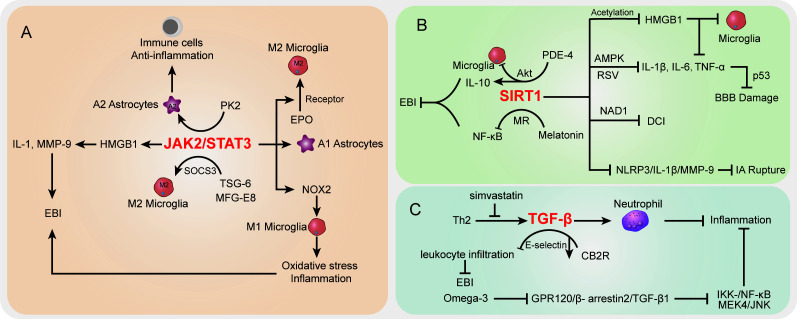
The specific role played by STAT **(A)**, SIRT1 **(B)**, and TGF-β **(C)** pathways in disease development following SAH.

In the SAH case, the activation of STAT-related signaling potentially contributed to morphological changes in cerebral arteries (207). The STAT pathway has been largely studied in vascular diseases (208). Recent studies demonstrated that STAT signaling was also involved in inflammation and immune cell balance during SAH (**Figure 3A**). JAK2/STAT3 signaling was regarded as an important inflammatory signaling pathway in mediating immune responses, which has a critical role in keeping the balance between pro-inflammation and anti-inflammation (209). For STAT3, a pivotal part of the STAT signaling cascade is known to regulate gene expression. The phosphorylation of STAT3 activated pro-inflammatory gene expression and influenced the pathologic progression of SAH (210). Among which, JAK2 is the essential component of STAT3 activation. Recently, An et al. reported that the activated JAK2/STAT3 cascade after mouse SAH was positively associated with pro-inflammatory molecular HMGB1 expression in both nucleus and cytoplasm (149), which subsequently promoted the pro-inflammatory cytokines like IL-1 and MMP-9 and contributed to EBI after SAH (150, 151). Simultaneously, many studies highlighted the HMGB1 function in brain injury and vasospasm, and inhibition of acetylation and release of HMGB1 paved a way to decrease inflammation after SAH (211, 212). It should be noted that the JAK2/STAT3 cascade was involved in immune cell microglial regulation after SAH. Pang et al. indicated that the JAK2/STAT3 cascade acted as the upstream of NADPH oxidase 2 (NOX2) expression in M1 microglia, which is the basis for oxidative stress and inflammatory cytokines in a SAH mouse model (152). Inhibition of the JAK2/STAT3/NOX2 cascade significantly suppressed the M1 microglial activation, subsequently improving the oxidative stress and inflammation. Moreover, targeting the STAT3 pathway showed potential to prevent BBB disruption following SAH (213) and exerted neuroprotective effects (214, 215). On the other hand, prokineticin 2 (PK2) was demonstrated to promote an anti-inflammatory A2 astrocytic phenotype and prevent neuronal injury (153). Ma et al. indicated that the effect of PK2 on the formation of A2 astrocytes of SAH was linked to STAT3 phosphorylation (154). Accumulation of A2 astrocytes potentially improved the immune cell, BBB, and neuron damage after SAH (216). Thus, the activated PK2/STAT3 cascade might promote the A2 astrocytes and improve the immune and inflammation environment to alleviate EBI after SAH (154). On the contrary, in the A1 pro-inflammatory astrocytic phenotype, activated STAT3 after SAH was deemed to be responsible for A1 activity, whereas the inhibition of STAT3 significantly abolished the astrocytic A1 polarization (217). The overactivation of STAT3 in A1 astrocytes is detrimental during SAH (217).

Interestingly, the activated JAK2/STAT3 cascade represented a severe condition after SAH, whereas its phosphorylation was observed to promote microglial M2 polarization and alleviate inflammation (155). Among which, the erythropoietin (EPO) treatment SAH model has upregulated EPO receptor (EPOR) expression along with the JAK2/STAT3 cascade to enhance the M2 polarization, whereas interfering with any of the above node will abolish the polarization process (155). Thus, the EPOR/JAK2/STAT3 cascade plays an important role in microglial functions and EBI after SAH. Furthermore, in microglial polarization, the SAH-protective molecule TNF-stimulated gene-6 (TSG-6) was deemed to play an important role in anti-inflammatory M2 phenotype transformation *via* the SOCS3/STAT3 cascade, wherein TSG-6 could decrease the STAT3 expression and increase SOCS3 expression (156). The TSG-6 protective effects in immune cell infiltration and inflammation have been wildly studied in the brain, especially the function in inhibiting the activation of microglia/macrophages (218). Recently, Gao et al. indicated that the interaction between milk fat globule-epidermal growth factor-8 (MFG-E8) and integrin β3 receptor could stimulate the SOCS3/STAT3 cascade then participate in the microglial M2 polarization and relieve the neuroinflammation after SAH (219). Of note, the STAT3 absence is the core to trigger the microglial morphological polarization process after SAH (220). The low abundance of TSG-6 will lead to the attenuated innate immunity response and elevate M1 microglia after SAH concomitant with inflammation and poor outcomes (156). Herein, the different cascades might contribute to distinct results after SAH and the therapeutic strategies targeting the STAT pathway should be carefully considered.

### The SIRT1 signaling pathway

SIRT1 is a class III histone deacetylase that controls a number of physiological processes, such as DNA damage repair, oxidative stress, inflammation, energy consumption, and cell death (221, 222). SIRT1 activity is dependent on and adjusted by nicotinamide adenine dinucleotide (NAD1) (223) (**Figure 3B**). NF-κB, p53, nuclear factor (erythroid-derived 2)-like 2 (Nrf2), forkhead box o (FOXO), hypoxia-inducible factors (HIFs), and liver X receptor (LXR) are histone and non-histone substrates that SIRT1 deacetylates (224, 225). The increased expression of SIRT1 has been reported to have a neuroprotective effect on brain edema and endogenous protection against DCI after SAH, as well as inducing the attenuation of neurovascular dysfunction following SAH (226–228), and the p53 pathway regulated by endogenous SIRT1 can crucially affect BBB permeability and brain edema after SAH (226). Concomitant with FOXO1, NF-кB, and p53 decreased acetylation, activation of SIRT1 pathways after SAH markedly reduced the levels of IL-1β, IL-6, and TNF-α; decreased Bax and cleaved caspase-3 levels and microglial activation; and increased Bcl-2 expression (229, 230). In EBI after SAH, pro-inflammatory cytokines (IL-1β, IL-6, and TNF-α) and neural apoptosis were also suppressed by resveratrol (RSV) *via* the AMPK/SIRT1 cascade (157). Meanwhile, melatonin has the ability to downregulate Ac-NF-κB and Bax expression and upregulate SIRT1 expression, suggesting that melatonin improved EBI following SAH through the melatonin receptor (MR)/SIRT1/NF-κB signaling pathway (158). Phosphodiesterase-4 (PDE-4) is crucial in a variety of injuries to the CNS, and PDE4 inhibition can inhibit neuronal apoptosis through the SIRT1/Akt pathway and ultimately protect rats from SAH-induced EBI (159). As a PDE4 inhibitor, rolipram significantly enhanced SIRT1 expression, whereas NF-κB activation is repressed in EBI after SAH. Mechanically, rolipram can upregulate protective cytokine IL-10 expression and inhibit pro-inflammatory cytokine (TNF-α, IL-1ß, and IL-6) expression as well as downregulate microglial activation (160). Moreover, the robust cerebral inflammation following SAH was linked to a considerable activation of the HMGB1/NF-kB pathway (14, 161). Accumulating evidence has shown that SIRT1 regulates HMGB1 hyperacetylation and suppresses HMGB1 translocation release (161). Zhang et al. indicated that enhanced SIRT1 expression can inhibit the inflammatory response mediated by HMGB1/NF-KB activation after SAH. As a selective SIRT1 inhibitor, ex527 reversed berberine-induced SIRT1 activation and attenuated berberine anti-inflammatory and neuroprotective effects on SAH, as illustrated by upregulated TNF-a, IL-1β, IL-6, and ICAM-1 release and microglial activation (161). Han and colleagues reported that oleanolic acid (OA) enhanced the expression of SIRT1 rather than suppressed the JAK/STAT3 pathway to lower the acetylation level of HMGB1. OA displays an anti-inflammatory effect by regulating TLR4, TNF-α, IL-1β, and NF-κB expression *via* SIRT1 signaling. HMGB1 is mostly expressed in neurons in EBI after SAH, which is associated with apoptosis, whereas HMGB1 is primarily expressed in microglia in DCI following SAH, which is associated with immunological activation (162). Moreover, as a multiprotein oligomer, the nucleotide-binding oligomerization domain–like receptor family pyrin domain–containing 3 (NLRP3) inflammasome is responsible for inflammatory response activation, which can promote IL-1β maturation and induce IL-1β release, ultimately leading to inflammation and tissue damage (231). More recently, in an aneurysm model under estrogen-deficient conditions, ERα and SIRT1 depletion may promote the activation of the NLRP3/IL-1β/MMP-9 pathway and enhance intracranial aneurysm rupture leading to SAH (163). Overall, targeting the SIRT1 pathway is a promising method to attenuate EBI and DCI after SAH *via* regulating inflammatory response.

### The TGF-β signaling pathway

Transforming growth factor (TGF)-β1 signaling plays an important regulatory role in endothelial cell differentiation, maintaining vascular wall integrity and the vascular network (232). The cortical and brainstem levels of TGF-β1 after SAH were remarkably enhanced in rats with the high-dose simvastatin group, which also repressed immunosuppressive cytokine TGF-β1 expression by lymphocytes and IL-1β expression post-SAH (233) (**Figure 3C**). Simvastatin triggers a Th2 immunological transition in these animals, and infiltrating Th2 cells are to blame for the observed rise in TGF-β1 production in the brain after therapy, ultimately providing neuroprotection against the neurological impairment following SAH (233). In addition, TGF-β1 can also inhibit neutrophil recruitment by reducing endothelial E-selectin expression (234). Cannabinoid-type 2 receptor (CB2R) agonism is reported to downregulate neuroinflammation (164). Fujii and colleagues suggested that CB2R stimulation prevents leukocyte infiltration into the brain by upregulating TGF-β1 and downregulating E-selectin, which protects the BBB after SAH and reduces neurological outcomes and brain edema (164). Meanwhile, omega-3 fatty acids are also able to exert effective anti-inflammatory effects through the G protein-coupled receptor 120 (GPR120) signaling pathway (165). Omega-3 fatty acids inhibited SAH-mediated inflammatory responses and apoptosis by the GPR120/β-arrestin2/TGF-β1 binding protein-1 (TAK1) anti-inflammatory pathway, eventually suppressing IKK-/NF-κB and MEK4/JNK downstream pathways (166), whereas fingolimod (FTY720), an immunomodulatory agent, enhanced Tregs and attenuated NKs in SAH mice treated with fingolimod after 3 days. Inflammatory cytokine IL-6 and TNF-α expressions were also decreased, whereas IL-10 and TGF-β1 were upregulated in serum with fingolimod post-SAH (235, 236). In summary, further research into drugs capable of modulating the TGF-1β pathway may provide new ideas for improving the post-SAH inflammatory response.

### The mTOR signaling pathway

**Figure 4:**
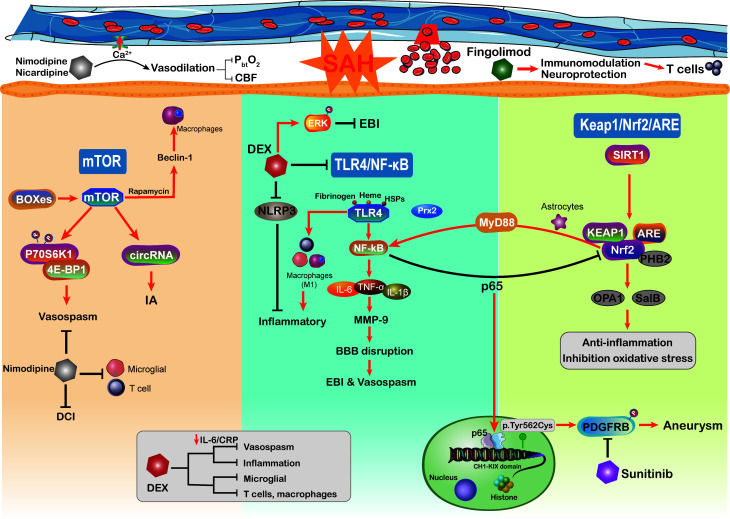
Schematic diagram of the mTOR, TLR4/NF-kB, Keap1/Nrf2/ARE signaling pathways and related therapy strategies in the pathophysiology of subarachnoid hemorrhage. CBF, cerebral blood flow; DEX, dexmedetomidine; CRP, C-reactive protein.

The mechanisms underlying poor prognosis following SAH are complex and multifactorial. The mammalian target of rapamycin (mTOR) is an atypical serine/threonine kinase involved in regulating major cellular functions, including growth, proliferation, survival, and protein synthesis (237). Through reducing excessive mitochondrial fission, mTOR inhibition protects against neuronal damage in EBI following SAH, indicating that mTOR activation additionally aggravated the neuronal and mitochondrial injury (238, 239) (**Figure 4**). As the core of the pathway–pathway interaction network, mTOR signaling is also associated with genes related to intracranial aneurysms (240). Circular RNAs (circRNAs) are closely related to many vascular diseases (241). Of note, by regulating the mTOR signaling pathway, circRNAs have been implicated in the formation of intracranial aneurysms (240). Moreover, mTOR has the ability to shape the immune system like immune cell migration, cytokine generation, antigen presentation, and macrophage polarization, further influencing the immune and inflammatory responses (242). On the other hand, previous studies have demonstrated that the mTOR signaling pathway plays a vital role in cerebral vasospasm following SAH (243). The increased levels of mTOR, P70S6K1, and 4E-BP1 (167) in basilar arteries were significantly associated with SAH and potentially mediated the activation of cerebral vasospasm. As a member of the PI3K family, mTOR orchestrates the phosphorylation of key downstream proteins P70S6K1 and 4E-BP1, both of which promote the proliferation of key vasculature wall cells (168). The mTOR/P70S6K1/4E-BP1 signaling pathway is significantly activated following SAH injury, and inhibition of mTOR is implicated as an attractive potential therapeutic strategy for vasospasm following SAH (243). Intriguingly, in a mouse brain ischemia model, inhibition of mTOR upstream suppressor PTEN observed that mTOR activation was directly involved in cortical neuron proliferation and enhanced neuronal axon densities (244). The mTOR activation improved long-term functional recovery after stroke rather than the acute phase, which may be beneficial for improving DCI after SAH (244). Moreover, the delayed cerebral vasospasm caused by bilirubin oxidation end products (BOXes) may have contributed to neurological impairment (167, 245). The end products of heme metabolism Z-BOX B significantly upregulated the phosphorylation of Akt, mTOR, and p70S6K, whereas rapamycin was able to counteract Z-BOX B’s effects. Recently, in a CoCl2-induced oxidative neuronal injury model, Z-BOX B dramatically reversed the hypoxia-induced neuronal injuries and stopped the apoptosis of primary cortical neurons through the Akt/mTOR/p70S6K signaling pathway (246). Meanwhile, the rapamycin specificity inhibited the expression of mTOR and upregulated the beclin-1 level to improve neuroprotective effects in ischemia reperfusion injury (169), where beclin-1 will stimulate the macrophage autophagy in the brain (247, 248). These data underscore the idea that targeting the mTOR signaling pathway can efficiently prevent macrophage function and suppress neuroinflammation in SAH patients (249). Hence, we need more studies to further confirm that targeting the mTOR signaling pathway modulates neuroinflammation after SAH.

### The TLR4/NF-κB signaling pathway

The TLR4/NF-κB signaling pathway plays an important role in the secretion of inflammatory factors such as IL-1β, TNF-α, and IL-6, which has been proved to be involved in the EBI following SAH (86, 250). TLRs are a family of receptors that play an essential role in brain innate immunity and inflammatory responses (251, 252). TLR-4 activation potentially acts as a costimulatory molecule for T-cell activation, where the cytokines IFN-γ and IL-1 participate in this process within the brain immune microenvironment and constitute the neuroinflammation (253–255). NF-κB is a putative inflammation regulator in multiple pro-inflammatory functions (256), which influences the DNA transcription and immune response in aneurysm SAH (171). Pro-inflammatory factors TNF-α, IL-1β, COX, and MMP-9 gene expressions were reported to be regulated by NF-κB (119, 257). The inhibition of NF-κB may alleviate MCP-1-induced macrophages infiltrating inflammation and reduce aneurysm formation and rupture (170). The aneurysm wall is characterized by brain immune cell population alteration, such as NKs, T cells, mast cells, and macrophages (258). On the other hand, the expression of NF-κB inhibitor gene NFKBIA in SAH patients has an anti-inflammatory effect and is associated with apoptosis and neurotrophin signaling (171). Activated microglia were attributed to roles as antigen-presenting cells and respond to TLR-4, thus shaping the adaptive immune response in the neuroinflammation (259). The activation of the NF-κB cascade is associated with increased microglial and macrophage populations in aneurysm (260). Moreover, the activation of TLR-4/NF-κB signaling can transfer macrophage into the M1 phenotype (261, 262). In aneurysm, macrophages do not merely influence the post-SAH inflammatory responses but also are related to intracranial aneurysm formation and rupture (257). The upregulation of M1 macrophage population showed the ability to mediate inflammation and promote the risk of rupture (257). Immune cell dysregulation and inflammation represent the cornerstones of aneurysm SAH occurrence; it seems to be the promising therapeutic target to the aneurysm SAH.

Some physiological derangements such as raised intracranial pressure and global cerebral ischemia after SAH have been shown to be mediated by inflammation and oxidative stress (8, 263). Accumulating evidence indicated that inflammatory cascades are involved in EBI after aneurysm SAH, especially the vasospasm (263). TLR4 activation is modulated by a variety of endogenous ligands including ROS, fibrinogen, heme, and heat shock proteins, all of which will be released following SAH (264). Moreover, patients with SAH are reported to express higher levels of TLR4 on PB cells, which is related to worse functional recovery and more serious SAH (265). The TLR4/NF-κB signaling pathway partly participated in cerebral vasospasm COX-1 upregulation (264). Moreover, activation of TLR4 on mononuclear cells is closely associated with cerebral vasospasm and DCI after aneurysm SAH, resulting in worse neurological function recovery (265). The inhibition of TLR4/NF-κB signaling decreased the EBI and cerebral vasospasm *via* improving the MyD88- (early phase) and TRIF- (late phase) dependent inflammatory response, which also protected against DCI and prevented poor outcomes (266). The efficient mechanism of TLR4 expression in regulating NF-κB activation has become a focus of research. In a recent study, damage-associated molecule peroxiredoxin 2 (Prx2) can regulate TLR4 function on microglia and subsequently stimulate the TLR4/NF-κB pathway following aneurysm SAH (172). Furthermore, TLR4/NF-κB signaling pathway activation may be involved in the mechanism by which neuroinflammation is exacerbated by releasing a multitude of inflammatory factors, such as IL-6, IL-β, TNF-α, and CD86 (267). Targeting the TLR4/NF-κB cascade not only downregulates pro-inflammatory cytokines levels but also alleviates the number of macrophages, neutrophil infiltration, and cell death. There is a great deal of evidence showing that the development of SAH is correlated with TLR4/NF-κB pathway activation, and this pathway may be a potential therapeutic target (86). In recent years, inhibition of the NF-κB cascade can efficiently alleviate SAH-related EBI, where the novel drug Netrin-1 shows the ability to improve the neurological deficits and brain injury *via* the regulation of the NF-κB signaling pathway (268).

### The Keap1/Nrf2/ARE signaling pathway

Nuclear factor (erythroid-derived 2)-like 2 (Nrf2) is an essential transcription factor that regulates the antioxidative system, which reduces the progression of various oxidative stress-related disorders (269, 270). It binds to a specific DNA site, antioxidant response element (ARE), to regulate the transcription of detoxifying or antioxidant enzymes (271). Nrf2 and ARE are key modulators in reducing inflammatory damage and oxidative stress, both of which are involved in SAH (272). Nrf2 also downregulates haptoglobin (Hp), hemopexin, red blood cells, and hemoglobin (Hb) after SAH (273). The haptoglobin (Hp; α and β peptide chains) phenotype determines outcomes in SAH and binds to hemoglobin (Hb) *via* a strong extracellular interaction (274). Previously, within the p62 catalyzing after SAH, the oxidized intracellular redox sensor Keap1 has been proved to directly accelerate Nrf2 release and activation (275, 276). Thus, activation of the Keap1/Nrf2/ARE pathway by its inducer may reduce the inflammatory response and ameliorate EBI after SAH. A recent study has shown that deletion of Nrf2 was associated with an increased inflammatory response and cell death (238). Continuous activation of the NF-κB pathway induces pro-inflammatory cytokine production and exacerbates inflammation (238, 273). Moreover, some studies have indicated that there is crosstalk between Nrf2 and the NF-κB signaling pathway in inflammation and injury (277, 278), which may influence the innate immune cell function. The NF-κB p65 subunit suppresses the Keap1/Nrf2/ARE pathway at the transcriptional level through competitively acting on the local histone hypoacetylation CH1-KIX domain (173). Furthermore, stimulation of the Keap1/Nrf2/ARE pathway after SAH can significantly downregulate the inflammatory response and oxidative stress (279). In addition to NF-κB upregulation, downstream inflammatory cytokines such as TNF-α, IL-1β, IL-6, and MMP9 are upregulated in astrocytes after SAH (174). Activated astrocytes play an important role in the neuro-immune axis and have the ability to modulate intracranial innate and adaptive immune response, like T cells, Tregs, and macrophages (175).

As a result of ROS generation, mitochondrial dysfunction is also involved in the pathological mechanism of EBI following SAH (280). The dynamic processes of mitochondrial function are closely associated with the Keap1/Nrf2/ARE signaling pathway (7). A previous study of hepatocellular carcinoma suggested that the binding of Nrf2 and prohibitin 2 (PHB2) is required for efficient expression (281). PHB2, an inner mitochondrial membrane protein, is a crucial receptor related to mitochondrial function (176). The synergistic expression of the downstream protein optic atrophy 1 (OPA1) not merely protects nerves after SAH but also is involved in the Nrf2-mediated signaling pathway (282). Treatments with increased levels of Nrf2, PHB2, and OPA1 have shown the ability to attenuate EBI (7). Therefore, attributing to its interaction with PHB2 in mitochondrial dysfunction, the Keap1/Nrf2/ARE pathway may play an important role in pathological mechanism underlying SAH.

In terms of oxidative stress, oxidative damage amelioration is associated with suppression of ROS generation and superoxide dismutase activity (270). The Keap1/Nrf2/ARE pathway has been demonstrated to be an antioxidant target in the SAH model of oxidative stress responses (279). Numerous recent studies have shown that SIRT1 exerts potent antioxidant effects by enhancing the expression and activity of the Keap1/Nrf2/ARE pathway, which can improve SAH-induced oxidative damage (229, 270, 283, 284). In addition, the Nrf2-ARE cascade is activated in the brain after SAH to prevent the brain from EBI, which probably inhibits cerebral oxidative stress by inducing antioxidant and detoxifying enzymes (285). A study in a mouse model of SAH showed that Nrf2 expression is upregulated in the arteries as a compensatory mechanism (286). Similarly, in another early SAH mouse model, Nrf2 expression was increased in the cortex in a time-dependent (12, 24, and 48 h) manner compared with the expression observed in the control group (285). Nrf2 knockout significantly reversed the antioxidant effects of salvianolic acid B (SalB) in SAH-induced oxidative damage (270). To date, substantial evidence demonstrated that the Keap1/Nrf2/ARE pathway is activated in SAH. Taken together, these results suggest that the Keap1/Nrf2/ARE pathway can be a target for immune modulation and anti-inflammatory and antioxidative therapy after SAH.

## Multiple treatment strategies for SAH

The management of patients with aneurysmal SAH remains a highly demanding challenge in critical care medicine. According to the above immune cells and inflammatory regulation, several therapeutic and preventive drugs from current preclinical and animal experiments were discussed in our review (**Table 2**). We summarized drug types with great potential to influence the immune inflammatory regulation after aneurysmal SAH like dexmedetomidine, nicardipine, nimodipine, and fingolimod, as well as prophylactic drugs for development of aneurysms like sunitinib (**Figure 4**).

**Table 2:** Therapy strategies in aneurysm SAH.

Agent	Administration	SAH model	Clinical effect	Treatment mechanism	Reference
Nicardipine/nimodipine	Oral/injection	Patient	Vasodilatation	Anti-inflammation	(287)
			Increase CBF		(288, 289)
Melatonin	Injection	Mouse	Attenuate brain edema		(84)
Dexmedetomidine	Injection	Mouse	Alleviate inflammation	Suppressed TLR4/NF-κB pathway	(290)
			Vasodilatation		(86, 291)
			Improve neurological function		(165)
Fingolimod	Oral	Mouse	Improve neurological outcome	Immunomodulatory	(292, 293)
Sunitinib	Oral	Patient	Aneurysms inhibition	PDGFRB mutation target	(26, 294)

### Nicardipine and nimodipine

Retrospective and prospective studies have shown that intrathecal nicardipine can improve outcome, decrease angiographic vasospasm, and downregulate mean blood flow velocity in SAH. The analogue of calcium channel antagonist nicardipine, oral nimodipine, remains the only FDA-approved medication to improve aneurysmal SAH (295). Nimodipine is lipophilic and intersects with intact BBB to achieve bioavailability (296). The average bioavailability of oral nimodipine (45 mg/4 h) was only approximately 16% of the maximal plasma concentration 1 h after ingestion in SAH patients (287). Hepatic metabolism by cytochrome P450 may contribute to the low plasma concentration. For the T cells, nimodipine can inhibit the Ca^2+^ influx to suppress T-cell function and related inflammation (297). In addition to modulating the immune inflammatory response, nimodipine also suppressed the T-cell energy metabolism, proliferation, and Th1 differentiation (297), which played a potential role in shaping the immune responses after aneurysm SAH. The previous *in vitro* studies demonstrated that L-type calcium channel blocking has a positive role in microglial activation suppression and further contributes to neuroprotection and pro-inflammatory factor inhibition (298). However, the dearth of dependable evidence for nimodipine effect in the clinical SAH immune microenvironment still limited the horizon of its application. Generally, appropriate microglial activation in the early stage is essential for harmful substance clearance after SAH, whereas the hyperactivated microglial will aggravates brain damage and promotes the release of pro-inflammatory cytokines, chemokines, and cytotoxic substances (299). Targeting the T-cell and microglial activation might be a novel therapeutic alternative for nimodipine in aneurysm SAH.

Moreover, pleiotropic mechanisms of nimodipine activation are associated with L-type calcium channel-mediated vascular smooth muscle cell vasodilation (300). Nimodipine has been shown to be an efficient vasodilator *in vitro* and *in vivo*, resulting in improvement in the outcomes after aneurysmal SAH (288). The inhibition of calcium influx into the cellular compartment may downregulate smooth muscle contractility, thereby causing vasodilatation (301). As a result, oral nimodipine administration decreases average arterial blood pressure and cerebral perfusion pressure. Similarly, monitoring of physiologic parameters after nimodipine administration revealed decreased brain tissue oxygenation (P_bt_O_2_) and poor cerebral blood flow (CBF) in aneurysmal SAH patients. Different physiologic changes in the brain oxygenation and pressure indexes were improved by nimodipine administration (301, 302). Of note, intra-arterial and intravenous nimodipine therapy after aneurysmal SAH was reported to attenuate cerebral artery constriction and increase CBF (289, 303). Previous angiographic studies showed significant and immediate clinical improvement (reaching 75%) in macro-vasoconstriction in 50%–65% of SAH patients following intra-arterial administration of nimodipine (304). In addition, through regulating the pressure reactivity index (PRx), the risks of rebound ischemia in patients at risk of recurrent vasospasm can be minimized by infusion with intra-arterial nimodipine (301, 305, 306). Nimodipine can also enhance lymphatic system function and mitigate neurological defects and cerebral edema in SAH mice by activating the cAMP/PKA pathway (307). Thus, nimodipine is not merely involved in immune cell regulation but also has shown a great potential in improving the SAH relevant vascular lesion.

However, there are studies on the efficacy of intravenous or oral administration of nimodipine and its use as an adjunct treatment option in aneurysmal SAH patients. In current applications, nimodipine provides less than optimal efficacy and causes dose limitation in a number of SAH patients (287, 300). Among the different routes of delivery, lipid-based drug delivery systems have attracted increasing attention due to their solubility, bioavailability, and stability (308). Recently, local administration of vasoactive drugs (nimodipine) and prolonged-release pellets has been shown to reduce the incidence of cerebral vasospasm and delayed ischemic deficits after severe aneurysmal SAH (309, 310). Of note, nicardipine prolonged-release implants (NPRIs) formulated in copoly(lactic/glycolic acid) (PLGA) have been developed as an approach to local delivery (310, 311). NPRIs have a good safety and tolerability profile with no complications and no signs of neuronal toxicity in aneurysm-clipped patients (310). Moreover, nanotechnology development has revealed that different structural modifications presented with great potential to improve the drugs’ therapeutic effect. Among these, nanostructured lipid carrier lactoferrin-modified PEGylated NLC (Lf-NLC) exhibits a high loading content and uniform particle size biodistribution, which was designed and constructed for the efficient delivery of nimodipine in treating strokes in the brain (296). It was previously observed that epithelial cells overexpressing low-density lipoprotein (LDL) provide a unique opportunity for therapeutic agent delivery by Lf (312). By crossing the BBB, Lf-NLC can be internalized into cytoplasm *via* the Lf-receptor-related endocytosis pathway to deliver nimodipine to brain tissues (296). Moreover, nimodipine dose reduction or discontinuation influenced by arterial blood pressure is a frequent occurrence, which is also related to poor clinical outcome (295). In contrast, the multivariate analysis showed that full dosage of nimodipine decreased the risk of unfavorable clinical outcome (OR 0.895, *P* = 0.029). The chemical modification of nimodipine significantly increased the local concentration and decreased the adverse reaction. In these studies, a more favorable clinical outcome, decreased mortality, improved cerebral vasospasm, and lower delayed ischemic lesion incidences have been reported.

NicaPlant (BIT Pharma), a novel sustained nicardipine release system composed of a mixture of two completely degradable polymers, has been developed to provide pharmaceutical equivalence and improve ease of manufacturing compared with NPRIs. In a chronic cranial window model, the application of NicaPlant for more than 3 weeks, with a higher arterial vessel diameter due to vessel dilatation (21.6 ± 2.6 µm vs. 17.8 ± 1.5 µm in controls, *P* < 0.01 vs. the control group), was observed by using *in vivo* epifluorescence video microscopy (313). The active ingredients in NicaPlant do not stimulate local tissue reaction, vessel leakage, or the leukocyte–endothelial cell interaction, which improve the safety of this delivery system. Maintaining a stable blood drug concentration is important for SAH patients’ treatment. Therefore, NicaPlant showed a good safety and efficacy profile in aneurysmal SAH patients compared with NPRI and improved patient outcomes while avoiding systemic side effects (313)

More recently, the results of the randomized, open-label, phase I/IIa dose-escalation trial of NEWTON (Nimodipine micro particles to enhance recovery while reducing toxicity after SAH) showed good safety, tolerability, pharmacokinetics, and clinical effects in aneurysmal SAH (314). NEWTON has been shown to be a safe and well-tolerated nimodipine microparticular formulation with a significant reduction in systemic side effects when compared with oral nimodipine (315). Furthermore, patients treated with NEWTON showed obvious reductions in DCI (31% NEWTON vs. 61% enteral nimodipine) and the need for rescue therapy (24% vs. 56%) (314). A new phase 3 double-blind, double-dummy, randomized NEWTON trial in aneurysmal SAH patients is currently underway in 374 participants (NTC02790632). However, no public interim trial data are available in clinicaltrails.gov. This study design might be helpful for the construction of new drug carriers and reduced vasospasm after aneurysmal SAH.

### Dexmedetomidine

Dexmedetomidine (DEX), a highly selective α_2_ receptor agonist, showed protective effects in many neurological diseases, including inflammation inhibition and lower sympathetic activity (316). As a potent antioxidant and anti-inflammatory drug, recent studies have shown that DEX exerts a neuroprotective effect in traumatic brain injury (317, 318). One study demonstrated that post-SAH treatment with DEX attenuated disease-related damage through activation of the extracellular signal-regulated kinase (phospho-ERK) (319). DEX (25 µg/kg) administered for SAH obviously decreased neutrophil infiltration, microglial activation, and pro-inflammatory factor release and improved the neurological scores and tight-junction proteins (290). In the first 24 h after SAH, hyperactivated microglial and several brain blood immune cells (T cells, macrophages, and neutrophils) were significantly reduced by DEX, potentially providing neuroprotection for brain injury (290, 320). DEX relieves microglial pyroction in post-SAH EBI by activating the PI3K/Akt/GSK3β pathway and inhibits SAH-induced release of pro-inflammatory cytokines (321). In terms of neuroprotection, this study also showed that DEX alleviated SAH-induced neuroinflammation in the context of the NLRP3 inflammasome and inhibited the TLR4/NF-κB pathway. The NLRP3 inflammasome is the most common inflammasome and is related to IL-1β and IL-18 secretion, which exacerbates the inflammatory response, apoptosis, and BBB disruption (80). Previous studies also demonstrated that inhibiting the NLRP3 inflammasome activation provides effective neuroprotection against EBI after SAH, suggesting that the NLRP3 inflammasome is a therapeutic target for SAH (322). In addition, there is compelling evidence indicating that TLR4/NF-κB pathway suppression may be a potential target for SAH therapy (86). A recent study showed that activation of the NLRP3 inflammasome occurs in two major steps. The first step involves microbial infection-related pathogen-associated molecular pattern (PAMP) signaling, and the second step involves inflammasome oligomerization and recruitment of apoptosis-associated speck-like protein (ASC) (290, 323). These processes convert pro-IL-1β and pro-IL-18 to mature IL-1β and IL-18. Meanwhile, DEX treatment significantly suppresses the expression of inflammatory factors IL-1β, TNF-α, and IL-6 in SAH (290). Thus, the anti-inflammatory effects of DEX in SAH may be mediated through suppression of the TLR4/NF-κB pathway and NLRP3 inflammasome activation.

The inflammatory response stimulated by SAH is involved in the process of vasospasm (19). The inflammatory response indicator, C-reactive protein (CRP), is produced by hepatocytes and is related to increased IL-6, both of which are closely linked to vasospasm after SAH (291, 324). Increased IL-6 and CRP levels after SAH may be a consequence of vasospasm. Conversely, DEX administration decreased the serum IL-6 and CRP levels in the SAH, eventually attenuating cerebral vasospasm and improving neurological deficit outcomes (324). DEX administration could attenuate SAH-induced vasospasm and improve the SAH rat activity score (325). Recently, DEX was also used as an adjunct therapy for brain injury and is related to sympathetic nervous system activity in the acute phase (326). Intriguingly, low-dose DEX contributed to a significant reduction in serum lactate levels 24 h after administration, which is associated with favorable clinical outcomes during the early phase in SAH patients (326, 327). However, the standard dosage response of DEX is associated with adverse events. Several studies demonstrated that serum lactate levels are regulated by multiple factors, and the sympathetic activity-related catecholamine release has been identified as a main factor in the acute phase of SAH (328, 329). Therefore, the DEX auxiliary role in SAH microenvironment cytokines and etiological factor regulation may ameliorate early-phase SAH symptoms.

### Fingolimod

Some pharmacological treatments for aneurysmal SAH are limited by the occurrence of hypotension (324). Consequently, novel and effective approaches for the treatment of aneurysmal SAH patients are urgently needed. As an oral immunomodulatory agent applied for the treatment of multiple sclerosis and common nervous inflammatory disorders, fingolimod (FTY720) was approved by the United States FDA in 2010 as a first-line drug for multiple sclerosis (236). FTY720 is a sphingosine-1-phosphate (S1P) analog, the therapeutic activity of which could be due to regulation of movement across the BBB, critical cellular processes, and lymphocyte subset migration (236, 330). Several studies have shown that FTY720 treatment of cerebral ischemia and hemorrhage improves brain edema, infarct size, stroke-related neuroinflammation, neuronal death, and clinical outcome (331, 332). Recently, an investigation of FTY720 for the aneurysmal SAH treatment in a rat model (292) showed that this intervention restricted intravascular leukocyte adhesion to pial venules and improved neurological outcomes. Simultaneously, by activating the PI3K/Akt/eNOS pathway, FTY720 was able to promote nitric oxide (NO) production, and the anti-apoptotic and anti-inflammatory effects of FTY720 can relieve cerebral vasospasm (333). In addition, FTY720 exhibited widespread distribution and long-term behavioral changes with a half-life of approximately 10 days (236, 331). FTY720 has also been shown to improve both innate and adaptive immunity in animal models. However, the immune-related molecular effects are species-specific (334). The different FTY720 regulatory mechanisms between mouse and human immune systems should be taken with more consideration. Current data indicate that various effects of FTY720 can influence critical elements of aneurysmal SAH, such as BBB permeability, neuroinflammation, and microvascular dysregulation (331, 335). Furthermore, FTY720 is known to retain CD4^+^/CD8^+^ T cells and central memory T cells in lymph nodes, which also has a partial effect on peripheral effector memory T cells and protection against infections (236). Previous studies have demonstrated that FTY720 can restrict circulating leukocytes and immune depression and improve outcome without increasing the risk of lung bacterial infections in a mouse model of cerebral ischemic stroke (293). There are, however, some similarities between aneurysmal SAH and transient cerebral ischemia (292). Accordingly, immunomodulation has emerged as a potential therapeutic strategy to alleviate brain injury and improve clinical outcome after aneurysmal SAH. Despite this, results obtained from experimental models require further investigation to confirm the long-term effects in humans.

### Sunitinib

Recent research has demonstrated that p.Tyr562Cys somatic genomic mutation (g.149505130T>C [GRCh37/hg19]; c.1685A>G) in the platelet-derived growth factor receptor β gene (PDGFRB) coding region might be a novel mechanism in the pathophysiology of intracranial aneurysms and suggest a potentially effective role of sunitinib in targeted therapy (26). Sunitinib, a targeted receptor agent used for tyrosine kinase inhibitors (TKIs) with anti-angiogenic and antitumor activity, is approved by the FDA for gastrointestinal stromal tumor (GIST) therapy (294). Overexpression or mutation studies have shown that the PDGF and PDGFR families play an important role in tumor cell growth and survival regulation. Moreover, PDGF functions mainly through two different receptor tyrosine kinases (336), PDGFR-α and PDGFR-β, and activates major signal transduction cascades such as the PI3K/Akt and phospholipase C-gamma pathways (337, 338). Sunitinib is a known inhibitor of PDGFR-β kinases (339). A recent study has shown that the p.R561C mutation in PDGFRB is associated with infantile myofibromatosis (340). In this study, sunitinib treatment significantly reduced PDGFRB phosphorylation and tumor cell proliferation without changing the phosphorylation of MEK1/2, ERK1/2, and several other protein kinases. Western blot analysis has shown that expression of the Tyr562Cys variant is consistent with higher basal levels of pPDGFRB, pSRC, pAKT, and pERK1/2, and PDGFRB phosphorylation can activate downstream signaling (26). PDGFRB phosphorylation contributed to cerebrovascular dilation and lesions, which is observed to be expressed in vascular smooth muscle cells (341). More recently, the individual with intracranial aneurysm was observed to have PDGFRB alteration, where the vascular complications like SAH may be associated with PDGFRB hyperactivation (342, 343). Interestingly, sunitinib-mediated inhibition of PDGFRB phosphorylation in intracranial aneurysms patients with the (p.Tyr562Cys) variation is more significant compared those with wild-type PDGFRB, which provides the potential to prevent intracranial aneurysm formation and rupture (26). However, the p.Asp850Tyr variation exhibited marked resistance to sunitinib under the same conditions. Therefore, appropriate sunitinib intervention in the early diagnosis of candidate variation and/or intracranial aneurysms should be taken into consideration. A similar study in abdominal aortic aneurysm demonstrated that PDGFRB imatinib inhibition obviously alleviated the deteriorated aneurysm (344). The identification sunitinib to PDGFRB phosphorylation inhibition provides a novel avenue for target therapeutic strategies in intracranial aneurysm and potentially prevents aneurysm-related malignant complications like SAH.

## Conclusions and perspectives

Intracranial aneurysm SAH is a devastating disease with a high fate ratio and limited prevention and treatment approaches. Accumulating evidence suggests that immune inflammatory responses, like different immune cells and inflammatory factors, potentially contribute to aneurysm SAH pathological events, such as immunosuppression, infection, cerebral vasospasm, EBI, and DCI. Moreover, the crosstalk between immune cells and inflammation regulation mechanism in the occurrence and development of aneurysm SAH cannot be ignored, especially the microenvironment changes and brain injury. Interconnected inflammation and immune cells, placed in the vicinity of the SAH region, elicited the pathogenic generation of molecules (IL-1, IL-6, TNF-α, MMPs, NLRP3) and have a critical role in local immune function deterioration. Importantly, the immunopathogenesis of SAH is also characterized by different signaling pathways, wherein compelling evidence revealed their functions in inflammation and immune cell regulation. The PI3K/Akt, ERK, STAT, and SIRT1 cascades seem to have an essential role in microglial and astrocyte-related immune regulation and inflammation response in SAH. Meanwhile, the HIF-α, mTOR, and TLR4/NF-κB cascades have been linked to macrophage immune regulation and brain injury following SAH and are possible to be the promising therapeutic targets. Distinguishing the mechanisms of immune suppression and hyperactivation will facilitate our understanding of personalized aneurysmal SAH treatment. However, due to a controversial signaling interaction between inflammation and immune cells and complex SAH pathological conditions, the underlying regulation mechanisms are still largely unknown. The pros and cons function of immune inflammatory modulation signaling activation in aneurysm SAH and neuroprotection should be further elucidated.

Therapeutic strategies targeting immune inflammation regulation showed a promising future in some preclinical studies. Aneurysmal SAH carries a high mortality and requires emergency treatment. However, there is still no robust evidence that anti-immune/inflammatory treatment can be apply to aneurysm SAH patients. Nimodipine targeting the calcium channel and/or cAMP/PKA pathway and its optimized chemical modifications improved aneurysm SAH therapy effects and decreased the adverse reaction and exhibited the ability for immune cell regulation. Through intervention of pathways like PI3K/Akt pathway, DEX-modulated ERK and TLR4/NF-κB cascade, and FTY720-related immune inflammation regulation, aneurysm SAH was likely to be prevented from more severe development. These available treatment options broaden the current horizons for aneurysm SAH therapy. However, heterogeneity of the aneurysm SAH immune inflammation and controversial drug-regulating mechanisms limited the clinical effects. The efficacy and safety of these drug and derivatives require further exploration. On the other hand, the drugs that prevent patients from aneurysm rupture and SAH should be another favorable direction. Understanding the basis of aneurysm development and rupture is important for early diagnosis and intervention. Although the aneurysm development and rupture development are still poorly understood, the immune inflammatory and genetic factors have non-negligible roles for SAH. Of note, genomic variations associated with aneurysm formation and rupture provide potential target treatment strategies for aneurysm SAH patients, such as sunitinib targeting the PDGFRB variant. The early aneurysm diagnosis and management showed great potential to reduce the harmful events. Taken together, further study is necessary to clarifying the immune and inflammatory regulation mechanisms, thus developing innovative drugs and target/systematic therapy strategies to improve clinical outcomes.

## Author contributions

JJ and JD conceived and drafted the manuscript. LD performed all literature searches. WX prepared and adjusted the figures. QZ and XP gave the concepts of the manuscript. QZ and XP approved the version to be submitted. All authors contributed to the article and approved the submitted version.

## Conflict of interest

The authors declare that the research was conducted in the absence of any commercial or financial relationships that could be construed as a potential conflict of interest.

## Publisher’s note

All claims expressed in this article are solely those of the authors and do not necessarily represent those of their affiliated organizations, or those of the publisher, the editors and the reviewers. Any product that may be evaluated in this article, or claim that may be made by its manufacturer, is not guaranteed or endorsed by the publisher.
